# Collateral Impact of Community-Directed Treatment with Ivermectin (CDTI) for Onchocerciasis on Parasitological Indicators of *Loa loa* Infection

**DOI:** 10.3390/pathogens9121043

**Published:** 2020-12-12

**Authors:** Hugues C. Nana-Djeunga, Cédric G. Lenou-Nanga, Cyrille Donfo-Azafack, Linda Djune-Yemeli, Floribert Fossuo-Thotchum, André Domche, Arsel V. Litchou-Tchuinang, Jean Bopda, Stève Mbickmen-Tchana, Thérèse Nkoa, Véronique Penlap, Francine Ntoumi, Joseph Kamgno

**Affiliations:** 1 Centre for Research on Filariasis and Other Tropical Diseases, P.O. Box 5797, Yaoundé, Cameroon; lenoucedric@gmail.com (C.G.L.-N.); cyrilledo4000@gmail.com (C.D.-A.); djune.linda@gmail.com (L.D.-Y.); floribertf@yahoo.com (F.F.-T.); domche_andre@yahoo.fr (A.D.); larvadel@gmail.com (A.V.L.-T.); bopda@crfilmt.org (J.B.); mbickmen@crfilmt.org (S.M.-T.); 2 Ministry of Public Health, Yaoundé, Cameroon; thereseparasitologie@yahoo.com; 3 Department of Biochemistry, Faculty of Science, University of Yaoundé 1, P.O. Box 812, Yaoundé, Cameroon; v.penlap@yahoo.fr; 4 Fondation Congolaise pour la Recherche Médicale (FCRM), Brazzaville CG-BZV, Republic of the Congo; fntoumi@fcrm-congo.com; 5 Faculty of Science and Technology, Marien Ngouabi University, P.O. Box 69, Brazzaville, Republic of the Congo; 6 Department of Public Health, Faculty of Medicine and Biomedical Sciences, University of Yaoundé 1, P.O. Box 1364, Yaoundé, Cameroon

**Keywords:** onchocerciasis, CDTI, loiasis, collateral impact, Cameroon

## Abstract

Ivermectin (IVM) is a broad spectrum endectocide whose initial indication was onchocerciasis. Although loiasis is not among its indications, IVM also exhibits antiparasitic activity against *Loa loa*. IVM-based preventive chemotherapies (PCs), so-called community-directed treatment with ivermectin (CDTI), have led to the interruption of transmission of onchocerciasis in some foci. A cross-sectional study was conducted in the Yabassi Health District where CDTI have been implemented since 20 years to fight onchocerciasis. All volunteers aged ≥ 5 years underwent daytime calibrated thick blood smears to search for *L. loa* microfilariae (mf). The prevalence of loiasis was 3.7% (95% CI: 2.2–6.2), significantly lower than its baseline prevalence (12.4%; 95% CI: 10.1–15.2; Chi-Square = 21.4; df = 1; *p* < 0.0001). Similarly, the microfilarial density was significantly low (mean = 1.8 mf/mL; SD = 13.6; max = 73,600) compared to baseline microfilarial density (mean = 839.3 mf/mL; SD = 6447.1; max = 130,840; Wilcoxon W = 179,904.5; *p* < 0.0001). This study revealed that the endemicity level of loiasis was significantly low compared to its baseline value, indicating a significant impact of IVM-based PC on this filarial disease. However, transmission is still ongoing, and heavily infected individuals are still found in communities, supporting why some individuals are still experiencing severe adverse events despite > 2 decades of CDTI in this Health District.

## 1. Introduction

Loiasis or African eye worm is a vector-borne disease caused by a parasitic nematode, *Loa loa*, transmitted by a tabanid belonging to the genus *Chrysops* that colonizes forested areas of West and Central Africa, considered as high-risk for loiasis, where over 14 million people currently reside [1,2]. Infection with *L. loa* manifests itself in human populations by pruritus and two main clinical signs, the migration of adult worms under the bulbar conjunctiva, and migratory edema commonly known as Calabar swelling. Apart from these clinical signs, which are considered benign though representing only nuisance for infected individuals, several studies have reported an association of *L. loa* infection with renal, cardiac, ocular and neurological complications [3], and excess mortality [4], thus giving a broader picture to the burden of loiasis [5]. Given the up to known accepted benign nature of this disease, no control program currently exists to fight this filarial disease.

Although no control program to fight loiasis currently exists, a number of anthelminthics, including IVM, are known to be active against *L. loa* [3]. Indeed, IVM has been registered in 1987 for human onchocerciasis control, and in the late 1990s, in combination with albendazole (ALB), for lymphatic filariasis elimination [6,7,8,9]. The community-directed treatment with ivermectin (CDTI) was adopted in 1997 by the African Program for Onchocerciasis Control (APOC) as its core strategy to fight onchocerciasis [10,11,12]. These control efforts have led to substantial reductions in the prevalence of infection, and the transmission of river blindness has been successfully interrupted by long-term mass IVM administration in some limited foci in Africa [13,14,15,16,17].

In Central and West Africa, there is a considerable overlap in the distribution areas of onchocerciasis and loiasis [1,18,19]. In these coendemic settings, it was established that individuals heavily infected with *Loa loa* (>30,000 microfilariae per milliliter of blood) can develop serious adverse events (SAEs) with sometimes neurological involvement and fatal outcomes when treated with IVM as part of preventive chemotherapy (PC) against onchocerciasis [20]. Since it was accepted that the benefit of IVM-based PC on onchocerciasis burden, especially in meso- and hyperendemic settings, outweigh the risk of SAEs, CDTI was recommended by the World Health Organization (WHO) in areas where loiasis was coendemic, though a surveillance system was highly needed to promptly manage potential SAE cases [21].

Since IVM was proven to be highly effective against loiasis, it is most likely that the post-CDTI impact observed with onchocerciasis [22,23] will be similar with loiasis in coendemic settings, as a collateral benefit of the long-term use of IVM and/or drug combinations (IVM and ALB). To date, data on the impact of CDTI on *L. loa* transmission are unfortunately scanty and those available are controversial. While some exhibited a significant decrease in the prevalence and intensity of *L. loa* infection both in human and vector populations [24,25], others support ongoing transmission with higher or substantially unchanged entomological indices despite 15 years of annual CDTI [26]. In this context of scarcity and controversy in available data, the present study aimed to assess the long-term collateral impact of CDTI against onchocerciasis on prevalence and intensity of *L. loa* infection.

## 2. Results

A total of 376 individuals accepted to participate in this survey and were examined for loiasis microfilaremia and attributable clinical signs. The sex ratio (male/female) was female biased (0.8) (Chi-square = 10.3; df = 1; *p* = 0.0013), and age of participants ranged from 5 to 90 years old (median age: 32 years old; interquartile range (IQR) = 15–53 years old; Table 1).

### 2.1. Prevalence and Intensity of Loa loa Infection

The prevalence of *L. loa* infection was 3.7% (95% CI: 2.0–6.2%) in the six communities of the Yabassi Health District, varying from 0.0% (95% CI: 0.0–3.3%) to 8.6% (95% CI: 3.7–18.6%) across communities (Table 1). *Loa loa* infection rates were similar between communities (Chi-square = 6.611; df = 5; *p* = 0.297), genders (Chi-square = 0.001; df = 1; *p* = 0.920) and age groups (Chi-square = 4.33; df = 3; *p* = 0.228). The mean microfilarial density was 1.8 mf/mL (13.6) in the Yabassi Health District (Table 2) and was similar between communities (chi-squared = 7.164; df = 5, *p* = 0.209) and across age groups (*p* = 0.4454). An increasing trend was observed in the microfilarial densities among positive cases according to age of enrollees (r = 0.12; *p* = 0.023).

### 2.2. Clinical Signs Attributable to Loiasis

None of the individuals examined presented with either Calabar swelling or migration of adult worm under the bulbar conjunctiva. Only a few participants (6.1%) declared suffering from pruritus. Although similar across age groups (*p* = 0.293) and between genders (*p* = 0.779), the proportion of enrollees suffering from pruritus was significantly lower in the community Yabassi compared to the other communities (Fisher exact test: *p* = 0.03289).

### 2.3. Adherence to Ivermectin Yearly Mass Distributions

Among the interviewees, 61.2% declared that they have taken IVM every year during the past five years (and were considered as fully compliers), but up to 14.4% reported never swallowed IVM during the past five years (and were considered as systematic non compliers). The proportions of fully compliers (individuals who swallowed IVM during the last five rounds of treatment) were significantly higher in communities Nkogmalan, Yabassi and Ndogpo (*p* < 0.04), but inversely the proportions of systematic non compliers were significantly lower in these communities (Supplementary Materials, Table S1).

### 2.4. Eighteen-Year Trends in Prevalence and Intensity of Loa loa Infection

A significant decrease was observed in the overall prevalence of loiasis, shifting from 12.4% (95% CI: 9.9–15.2%) in 2000 (baseline) to 3.7% (95% CI: 2.1–6.3%) in 2018 (post-IVM follow-up visit) (*p* < 0.001). The percentage difference was globally relatively high (−70.2%, indicative of a reduction in infection rates), ranging from 11.7% (slight increase) in the Ndogpo community to 100% (total clearance) in the Yabassi Community (Table 1). The percentage reductions in *L. loa* infection rates were higher among females (−72.9%) and lower among the youngest enrollees (−36.4%; Table 1).

**Figure 1:**
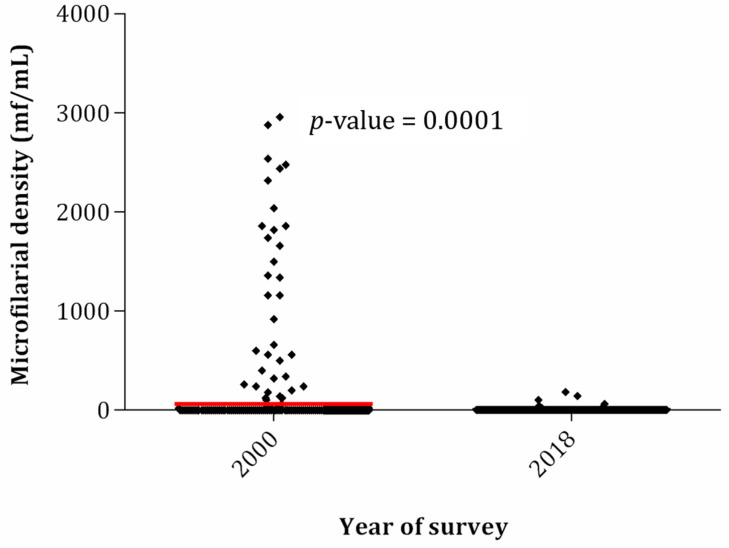
Trends in *Loa loa* microfilarial densities between 2000 (baseline) and 2018 (follow-up). For a better visualization of the plot, microfilaremia densities from 26 individuals (median: 8980 mf/mL; mean: 19,020 mf/mL; max: 130,840 mf/mL) in 2000 (baseline) and one individual (73,600 mf/mL) in 2018 (follow up) were considered as “outliers” and removed from the plotting.

Likewise infection rates, a significant decrease in microfilarial densities, from 839.3 (SD: 6447.1) to 1.8 mf/mL (SD: 13.6; Wilcoxon W = 179,904.5; *p* < 0.0001), was observed in the Yabassi Health District between 2000 (baseline) and 2018 (post-ivermectin follow-up visit), respectively (Table 2, Figure 1). The overall percentage reduction was very high (−99.8%), ranging from −70.9% in Ndogpo community to −100% in the community Yabassi. These percentage differences were similar among genders, but significantly low among the youngest individuals (−31.8%) compared to their elder counterparts (Table 2).

## 3. Discussion

The objective of this study was to assess the impact of 18 years of IVM-based preventive chemotherapy (PC) on the prevalence and intensity of *L. loa* infection in the Yabassi Health District in the Littoral Region, Cameroon.

The Yabassi Health District belongs to the Littoral CDTI project, and treatments with ivermectin have been initiated in that area since the years in the 2000s to fight onchocerciasis. Indeed, IVM is a macrocyclic lactone acting by killing almost 70–80% *L. loa* microfilariae within the first three days after a single dose of the drug, a total clearance being observed within 4–5 days in some individuals [27,28]. In addition to its principal effect (microfilaricidal), IVM also prevents for about three months the release of new microfilariae by adult female worms, so-called embryostatic effect. However, ivermectin is not adulticidal, though some macrofilaricidal efficacy has been described in the treatment of *O. volvulus*—a filarial parasite closely related to *L. loa*—after repeated doses of IVM [29]. Since *L. loa* adult worms have a long life (lifespan estimated up to 15–17 years) [30], repeated treatments with IVM are likely needed to interrupt the transmission of this filarial disease, the number of rounds of treatment remaining unknown. The long-term (18 years) implementation of CDTI in the Yabassi Health District likely explains the marked decrease observed in both prevalence and intensity of *L. loa* infection as was already observed elsewhere in Cameroon [25].

The percentage differences were less important for infection rates (−70.2%) compared to the intensity of infection (−99.8%), likely indicative of the fact that adult worms are life-long and producing microfilariae for their whole life. As such, repeated doses of IVM will reduce the productivity of adult worms and therefore the microfilarial densities, but prevalence will remain almost unchanged, especially during the first years/rounds of treatments. This observation supports why percentage differences are also low among the youngest individuals. The impact of IVM on parasitological indicators of *Loa loa* infection (prevalence and microfilarial density) was also found to be highly variable among communities (Table 1 and Table 2), most likely related to adherence to CDTI organized in this health district since almost two decades. Indeed, communities where the highest percentage reductions in *Loa loa* prevalence and microfilarial densities were in general found exhibiting a higher proportion of fully compliers and lower proportions of permanent non compliers. However, a completely different picture was observed between compliance to CDTI and prevalence and intensity of onchocerciasis in the community Ndogpo, suggesting that other drivers such as population structure (age and gender), migrations can also explain, even at a lesser extent, the long-term dynamics of onchocerciasis endemicity.

None of the patients interviewed remembered having experienced Calabar swelling and migration of adult worm under the bulbar conjunctiva, which are the most common clinical signs of loiasis used for rapid mapping of loiasis [31]. Indeed, it has been previously demonstrated that apart from its direct effect on parasites, IVM exhibits a beneficial effect on the clinical manifestations of loiasis, for example by preventing the reappearance of Calabar swelling for several months [32]. This likely suggests that these enrollees might have experienced these clinical signs a very long time ago and might be victims of memory bias. Only a few participants declared suffering from pruritus; although this can be likely due to the low level of *L. loa* microfilarial densities observed in the framework of this study, it is important to mention that this clinical manifestation is shared with other filarial diseases, and the Yabassi Health District is known to be endemic to onchocerciasis [33].

Despite the significantly high decreasing trends observed both in rates and intensities of infections, the transmission of the disease was still ongoing even though the endemicity level was quite low. This slight persistence of the disease can be explained by the presence in the communities of systematic non compliers, who can disseminate the disease and contribute to the persistence of the infection if infected, especially in a context where the chrysops vectors of *L. loa* are known to be highly competent [26,34]. Since there is no dedicated control program against loiasis because of its up to now accepted benign nature, efforts should be devoted to clarify the clinical impact and burden of loiasis so that the latter can be added to the WHO list of neglected tropical diseases. Importantly, some enrollees exhibited very high *L. loa* microfilarial densities and are therefore at risk of developing severe adverse events (SAEs) if treated with IVM. This suggests that despite multiple rounds of IVM, the risk of developing SAEs is still present, especially among those individuals receiving IVM for their first time or who have interrupted treatment for a while, whatever the reason. Onchocerciasis and lymphatic filariasis control or elimination programs should consider testing for loiasis before administering IVM to all those involving in CDTI for their first time or whiling to resume PC after a long time interruption, and this can be made possible by the recently developed “Test and Not Treat” procedure [35,36].

## 4. Materials and Methods

### 4.1. Ethics Approval and Consent to Participate

This study has been approved by the Institutional Review Board (IRB) of the Faculty of Medicine and Biomedical Sciences of the University of Yaoundé I (N°0097/UY1/FMSB/VDRC/CSF). After approval of the local administrative and traditional authorities, the objectives and schedules of the study were first explained to community leaders and to all eligible individuals. Written agreements were obtained from those who agreed to participate, under the discretion of community leaders. The approval of parents or legal guardians of minors was necessary before any procedure. An individual code (barcode) was attributed to each participant for anonymous data analysis.

### 4.2. Study Area and Population

This study was carried out in the Yabassi Health District (4°27′16″ N, 9°57′56″ E), located in the Nkam Division (Littoral Region, Cameroon), at 100 km north-east from Douala, the economic capital of Cameroon. According to the heath population denominator, the population of the Yabassi Health District was estimated at 14,532 inhabitants in 2017 [37]. The altitude of the area varies from 10–800 m, and the vegetation is mainly dense humid forest. Due to the intense hydrographic network (Nkam, Dibamba and Makombé) and the dense vegetation cover maintained by abundant precipitation, the relative humidity is high, thus favoring the development of the chrysops, vectors of *L. loa*. Agriculture is the main activity and is practiced by at least 60% of inhabitants.

### 4.3. Baseline Data and History of CDTI

Prior to the implementation of CDTI, baseline data of loiasis infection was collected in the study area in 2000. A total of 637 individuals were examined for loiasis, revealing an overall prevalence of 12.4% (95% CI: 10.1–15.2%) and a mean microfilarial density of 839.3 (standard deviation (SD): 6447.1) mf/mL (Table 1; Kamgno, Unpublished data). According to the National Onchocerciasis Control Program (NOCP), CDTI was launched in the Yabassi Health District in 1999 and since then, implemented at a yearly basis. At the outset of this study in 2018, up to 20 rounds of IVM mass administration had already been organized in this Health District, with therapeutic coverages globally higher than 80%.

### 4.4. Study Design

A cross-sectional survey was carried out in six communities of the Yabassi Health District in 2018. All individuals both males and females, aged ≥ 5 years old, either permanent residents who had lived continuously in the selected communities for the previous 5 years or migrants who had already lived for at least five years in the selected communities were eligible for the survey. The objectives and schedule of the study were clearly explained to all eligible individuals and those who agreed to participate in this study signed an informed consent form. All volunteers underwent a parasitological examination to search for *L. loa* microfilariae, a clinical examination to investigate clinical signs attributable to loiasis, and the history of CDTI and the migration of population was assessed to complement parasitological and clinical data.

### 4.5. Clinical Examination

During the medical consultation, all volunteers were interviewed about pruritus and a clinical examination was carried out to search for clinical signs of the disease (Calabar swelling and migration of adult worm under the bulbar conjunctiva). Subsequently, the history of the subconjunctival migration of the adult worm and Calabar swelling was assessed using a questionnaire adapted to the proven rapid assessment procedure for loiasis (RAPLOA) [31]. Briefly, participants were asked two questions: “Have you ever experienced or noticed worms moving along the white of the lower part of your eye?” to assess the experience of eye worm, and “Have you ever experienced swellings under the skin that changed position and disappeared?” to investigate the experience of Calabar swellings. Investigators presented pictures of these striking clinical signs to participants and were assisted by local guides to ease communication with villagers, especially illiterates.

### 4.6. Parasitological Examination

A parasitological examination consisting of a calibrated thick blood smear was performed from capillary blood sampling from the fingertip, about 10 months after the latest round of CDTI. This sampling was carried out by qualified personnel, under aseptic conditions using sterile and disposable materials, and between 10:00 am and 4:00 pm to take into account the diurnal periodicity of *L. loa* microfilariae [38]. Slides were examined under a light microscope at low magnification (×10 or ×40) by trained laboratory technicians. *L. loa* microfilaria were identified and counted and the results were expressed in microfilariae per milliliter of blood (mf/mL) [39].

### 4.7. History of Migration and Adherence to Mass Treatment

All enrollees were asked if they were natives or not of the visited community, and if non-natives, they were questioned on the number of years they had already spent in the community and on the communities visited before settling in the targeted community. In addition, compliance to the CDTI for the past 5 years was assessed by asking each participant whether he had swallowed IVM tablets during these treatment campaigns. A sample of the IVM tablets was presented to each of participant so that the answers did not relate to other mass campaigns.

### 4.8. Data Analysis

Data analysis was performed using the software R v3.5.2 (The R foundation for Statistical Computing, Vienna, Austria). Indeed, all relevant data for loiasis were entered using a purpose-built Microsoft Access database and subsequently exported into R software for statistical analyses. Microfilaremia and clinical signs prevalence were expressed as the percentage of infected or affected individuals among the total number of individuals examined. The intensity of infection was computed as the arithmetic mean microfilarial count, and the sampling fluctuations estimated using standard deviation (SD). Chi-square and McNemar tests were used to compare loiasis prevalence between genders, age groups and communities of the residence of participants. Mean intensities of infection were compared between genders, age groups and communities using Mann–Whitney and Kruskal–Wallis tests, and between years 2000 (baseline) and 2018 (follow-up) using the Wilcoxon ranked test. The threshold for significance was set at 5%.

## 5. Conclusions

The present study shows a significant impact of long-term IVM-based preventive chemotherapies (CDTI) on *L. loa* infection endemicity levels. Although the *L. loa* microfilarial prevalence and densities were quite low, an important proportion of systematic non-compliers and heavily infected individuals were recorded, suggesting that the risk for occurrence of SAEs remains possible despite almost two decades of CDTI, raising the interest on the identification of these at risk individuals before IVM administration.

## Figures and Tables

**Table 1:** Trends in prevalence of *Loa loa* infection between baseline (year 2000) and post-community-directed treatment with ivermectin (CDTI) follow-up (year 2018).

Variables	Baseline (Year 2000)		Follow-Up (Year 2018)		% Difference
	*N* Examined	*N* Infected (%)	*N* Examined	*N* Infected (%)	
**Genders**					
Female	282	50 (17.7)	210	8 (4.8)	−72.9
Male	355	29 (8.2)	166	6 (2.9)	−64.6
**Age Groups**					
5–20	45	1 (2.2)	147	2 (1.4)	−36.4
20–35	126	15 (11.9)	48	2 (4.2)	−64.7
35–50	153	21 (13.7)	68	3 (4.4)	−67.9
50–90	313	42 (13.4)	113	7 (6.2)	−53.7
**Communities**					
***Longtoka Health Area***					
Longtoka	114	13 (11.4)	59	2 (3.4)	−70.1
Ndogpo	39	3 (7.7)	58	5 (8.6)	11.7
Nkogmalan	84	14 (16.7)	45	2 (4.4)	−73.6
***Yabassi Centre Health Area***					
Bodiman	81	6 (7.4)	73	1 (1.4)	−81.0
Ndogbele	207	30 (14.5)	102	4 (3.9)	−73.1
Yabassi	112	13 (11.6)	39	0 (0.0)	−100
**Overall**	**637**	**79 (12.4)**	**376**	**14 (3.7)**	**−70.2**

*N*: number of participants.

**Table 2:** Trends in *Loa loa* microfilarial densities between baseline (year 2000) and post-CDTI follow-up (year 2018).

Variables	Baseline (Year 2000)		Follow Up (Year 2018)		% Reduction
	*N* Examined	Microfilarial Density (SD)	*N* Examined	Microfilarial Density (SD)	
**Genders**					
Female	282	1517.0 (9410.3)	210	1.7 (10.0)	−99.9
Male	355	301 (1930.2)	166	1.9 (15.9)	−99.9
**Age Groups**					
5–20	45	0.44 (3.0)	147	0.3 (2.3)	−31.8
20–35	126	1975.5 (12,737.5)	48	0.4 (2.9)	−99.9
35–50	153	610.5 (4169.1)	68	3.8 (20.8)	−99.4
50–90	313	614.3 (3249.4)	113	3.2 (18.4)	−99.5
**Communities**					
***Longtoka Health Area***					
Longtoka	114	754.0 (4223.1)	59	1.4 (8.2)	−99.8
Ndogpo	39	17.9 (91.2)	58	5.2 (22.7)	−70.9
Nkogmalan	84	1091.4 (3963.0)	45	0.9 (4.2)	−99.9
***Yabassi Centre Health Area***					
Bodiman	81	2.7 (12.6)	73	0.3 (2.3)	−88.9
Ndogbele	207	1449.2 (10,342.2)	102	2.4 (18.4)	−99.8
Yabassi	112	500.7 (2851.9)	39	0.0 (0.0)	−100.0
**Overall**	**637**	**839.3 (6447.1)**	**376**	**1.8 (13.6)**	**−99.8**

*N*: number of participants; SD: standard deviation; microfilarial density was expressed as the number of microfilariae per milliliter of blood (mf/mL).

## Supplementary Materials

The following are available online at https://www.mdpi.com/2076-0817/9/12/1043/s1, Table S1: Compliance to ivermectin mass distribution in different communities of the Yabassi Health District.

## Abbreviations

ALB	albendazole
CDTI	community-directed treatment with ivermectin
CI	confidence interval
IVM	ivermectin
mf/mL	microfilariae per milliliter of blood
mf	microfilariae
PC	preventive chemotherapy
SAE	severe adverse event
SD	standard deviation
