# Supplementary material for: Collateral Impact of Community-Directed Treatment with Ivermectin (CDTI) for Onchocerciasis on Parasitological Indicators of Loa loa Infection
Source: Pathogens. 2020 Dec 12;9(12):1043. doi: 10.3390/pathogens9121043 (PMC7764802; doi:10.3390/pathogens9121043)
Supplement: Supplementary file 1 [file pathogens-09-01043-s001.pdf]

Table S1. Compliance to ivermectin mass distribution in different communities of the Yabassi Health District

**Communities**	**No. fully compliers* (%)**	**No. partially compliers** (%)**	**No. systematic non compliers*** (%)**
Bodiman	32 (43.8)	28 (38.4)	13 (17.8)
Longtoka	30 (50.8)	18 (30.5)	11 (18.6)
Ndogbele	67 (65.7)	16 (15.7)	19 (18.6)
Ndogpo	39 (67.2)	15 (25.9)	4 (6.9)
Nkogmalan	35 (77.8)	6 (13.3)	4 (8.9)
Yabassi	27 (69.2)	9 (23.1)	3 (7.7)
**Total**	**231 (61.2)**	**93 (24.5)**	**54 (14.4)**

**Fully compliers are considered as individuals who swallowed ivermectin during the last five rounds of treatments; **Partially compliers are considered as those enrollees who swallowed ivermectin 1-4 times during the last five rounds of treatments; ***Systematic non compliers are considered as those participants who never swallow ivermectin during the last five rounds of treatments.*
